# Colorectal liver metastases patients prognostic assessment: prospects and limits of radiomics and radiogenomics

**DOI:** 10.1186/s13027-023-00495-x

**Published:** 2023-03-16

**Authors:** Vincenza Granata, Roberta Fusco, Sergio Venanzio Setola, Roberta Galdiero, Nicola Maggialetti, Renato Patrone, Alessandro Ottaiano, Guglielmo Nasti, Lucrezia Silvestro, Antonio Cassata, Francesca Grassi, Antonio Avallone, Francesco Izzo, Antonella Petrillo

**Affiliations:** 1 Division of Radiology, “Istituto Nazionale Tumori IRCCS Fondazione Pascale – IRCCS di Napoli”, Naples, Italy; 2 Medical Oncology Division, Igea SpA, Napoli, Italy; 3 Italian Society of Medical and Interventional Radiology (SIRM), SIRM Foundation, Via della Signora 2, Milan, 20122 Italy; 4 Department of Medical Science, Neuroscience and Sensory Organs (DSMBNOS), University of Bari “Aldo Moro”, Bari, 70124 Italy; 5 Division of Epatobiliary Surgical Oncology, Istituto Nazionale Tumori IRCCS Fondazione Pascale—IRCCS di Napoli, Naples, 80131 Italy; 6 Clinical Sperimental Abdominal Oncology Unit, Istituto Nazionale Tumori, IRCCS Fondazione G. Pascale, Napoli, 80131 Italy; 7 Division of Radiology, “Università degli Studi della Campania Luigi Vanvitelli”, Naples, 80138 Italy

**Keywords:** Liver metastasis, Radiomics, K-Ras mutation, Mucinous histological subtypes, Growth pattern

## Abstract

In this narrative review, we reported un up-to-date on the role of radiomics to assess prognostic features, which can impact on the liver metastases patient treatment choice. In the liver metastases patients, the possibility to assess mutational status (RAS or MSI), the tumor growth pattern and the histological subtype (NOS or mucinous) allows a better treatment selection to avoid unnecessary therapies. However, today, the detection of these features require an invasive approach. Recently, radiomics analysis application has improved rapidly, with a consequent growing interest in the oncological field. Radiomics analysis allows the textural characteristics assessment, which are correlated to biological data. This approach is captivating since it should allow to extract biological data from the radiological images, without invasive approach, so that to reduce costs and time, avoiding any risk for the patients. Several studies showed the ability of Radiomics to identify mutational status, tumor growth pattern and histological type in colorectal liver metastases. Although, radiomics analysis in a non-invasive and repeatable way, however features as the poor standardization and generalization of clinical studies results limit the translation of this analysis into clinical practice. Clear limits are data-quality control, reproducibility, repeatability, generalizability of results, and issues related to model overfitting.

## Background

There is no doubt that from a radiologist point of view, the liver is probably the most insidious target, given the considerable amount of benign and malignant processes that can involve it [1–8]. Moreover, different pathologies can be synchronous or metachronous, so radiologists dedicated to the study of the liver should be experts and collaborate with a multidisciplinary team [9–20]. In a such complex situation, the radiological management of liver metastases patient requires great skill considering, also, the different management phases [21–24], as in terms of the most suitable radiological tools to use [25–43], as in term of response assessment after different therapies [44–50].

In addition, the radiologist role has profoundly changed, having to answer increasingly critical questions. In fact, compared to the characterization and evaluation of lesion resectability, today a prognostic assessment is also required, for the identification of several features that can impact on the therapeutic choice [23–38].

Among metastatic patients, the overall survival (OS) is profoundly related to different features, such as the stage of the disease, the lesion mutational status as well as the patient physical condition and it has been reported that although about 70–75% of patients survive within 1 year, fewer than 20% outside 5 years [51]. The main treatment for unfit surgical resection lesions is systemic therapy, based on the combination of cytotoxic chemotherapies, biologic therapies and or immunotherapy. Several clinical experiments have proven that modifying treatments according to tumor molecular and pathologic profiling could improve OS. Genomic characteristics are a critical point since these allow to identify the therapies that should be efficient. It has been proven that about the 50% of patients with KRAS/NRAS/BRAF wild-type lesions, can be treated with monoclonal antibodies to the epithelial growth factor receptor (EGFR) in combination with conventional cytotoxic treatments, with an OS improvement of 2 to 4 months compared to chemotherapy alone [51]. So as, immunotherapy could be utilized as upfront treatment, in patients with microsatellite instability or mismatch repair deficiency, improving OS of 31.4 months [51].

In addition, directed therapy, including hepatectomy, radiofrequency or microwave ablation, and/or hepatic artery infusion chemotherapy (HAIC), can be associated with 5-year OS as high as 60% [52, 53]. The definition for resectability of liver metastases in recent years has developed to comprise any patient in whom all liver lesions can be surgically eradicated with negative margins (R0) and an adequate future liver remnant can be preserved [54, 55]. However, about 60% of patients will have recurrence in the liver even after a complete surgical removal of all primary liver metastases.

At this time, it is complicated to exactly predict clinical outcome considering patient and primary lesion features. Clinical risk scores of Fong and Nordlinger were usually adopted to assess clinical outcome in this context [56, 57]. However, the validity of these risk scores is doubted, since these are considering outcomes of patients in the 1990’s, when the approach to treat liver metastases patients was overall more conservative [56, 57]. Another pre-surgical score, the Genetic And Morphologic Evaluation (GAME) score was improved, but is still not utilized in clinical setting [58]. Biomarkers correlated to the outcome can help the patient management. To this end, several prognostic biomarkers have been proposed, mainly focused on clinic-pathological characteristics as KRAS and BRAF mutational status, histopathological features (mucinous), and surgical resection margin [59–67].

Recently, the idea that imaging studies contain a great quantity of data, in form of grey level patterns, which are imperceptible to the human eyes, has become more and more interesting [68–80]. These texture features, when correlated with clinical-pathological data and outcomes [81–93], theoretically allow diagnostic and prognostic assessment [94–115]. The assessment of textural characteristics, obtained by radiological images, which depend on mathematical analysis, as histogram analysis, is called radiomics [116–133]. This approach is captivating since it should allow to extract biological data from the radiological images [134–156], without invasive approach, so that to reduce costs and time, avoiding any risk for the patients. For several tumors, radiomics analysis has already demonstrated an accurate biological features evaluation [157–174].

In this narrative review, we reported un up-to-date on the role of radiomics to assess prognostic features, which can impact on the liver metastases patient treatment choice.

## RAS mutational status and radiomics

Advances in surgery and systemic therapy have improved the percentage of patients with liver metastases, which are technically resectable [175]. Although the number of technically resectable lesions was increasing, surgeons recognized the importance of tumor biology beyond technical resectability alone [175]. Even if the molecular studies initiated in the 2000s, only in 2013, Vauthey et al [176] demonstrated the prognostic role of molecular data (RAS mutation) in patients with colorectal liver metastases, so that RAS mutational status was routinely tested by determining the eligibility for anti-EGFR treatment. Consequently, Johns Hopkins Hospital (JHH) team [177] and The University of Texas MD Anderson Cancer Center team [178] combined RAS status with several clinic-pathologic features to develop the first two hybrid clinical and genetic risk scores. In addition, it has been demonstrated the negative effect of RAS mutations in patients re-treated with hepatectomy for recurrent liver metastases, so that RAS mutational status may impact on selection for a second hepatectomy [179].

KRAS, NRAS, and HRAS are the RAS oncogenes to encode a family of guanidine triphosphates (GTP)-adjusted switches [180–182]. KRAS gene is correlated to the colorectal cancer development and progression, representing an independent prognostic risk factor. Usually, the assessment of gene mutation status is based on the examination of specimens obtained from surgery or biopsy, which are expensive and invasive approaches. Since Lambin et al, first proposed the concept of Radiomics in 2012, this high-throughput, non-invasive strategy has been shown to provide addition data that can offer assistance for clinical decision making in several settings and different tumors [183–220]. Several previous studies have assessed the role of radiomics and RAS status in the primary colorectal lesions [221–223].

With regard to liver mutational status, the opportunity to compare RAS status and radiomic features provide significant advantages compare to quality evaluation, since this analysis allows a better patient selection for treatment, to predict response to therapies, discriminating favorable and unfavorable subsets of patients, including patients which could benefit from surgical resection [224–259]. Yang et al. [225] evaluated KRAS/NRAS/BRAF mutation in 117 untreated primary lesions (61 in the training and 56 in the validation set), obtaining 346 radiomics features from portal phase of CT studies. They demonstrated that radiomics features were significantly correlated with KRAS/NRAS/ BRAF mutation.

Lubner et al. [226] assessed texture features obtained from liver metastasis CT studies in 77 untreated patients, showing that entropy, mean positive pixels and standard deviation of medium filtration were correlated to tumor stage. In addition, skewness was negatively correlated to KRAS, while the coarse filtration entropy was correlated to OS.

Shi et al [227], in a multi-centric retrospective study, evaluated 159 untreated patients, which underwent CT studies. Radiomics features were obtained from the portal phase of the contrast studies. Seven machine learning algorithms were utilized to establish three scores based on the semantic, radiomics and the combination of both features. Two semantic and 851 radiomics features were used to predict the mutation status of RAS and BRAF using an artificial neural network method (ANN). They showed that this score can allow to distinguish wild type and mutant patients with an AUC of 0.95 in the training set and 0.79 in the validation set.

Granata et al [228] evaluated the association of RAS status and radiomics metrics by Contrast Enhanced (CE)-Magnetic Resonance Imaging (MRI). Significant results were obtained only for texture features using multivariate analysis, while the univariate analysis did not allow RAS status assessment.

## Microsatellite instability and radiomics

Several types of genomic instability can drive tumor initiation and development. The main frequent type in colorectal cancer (about 85%) is chromosomal instability, while microsatellite instability (MSI) is found in 15% of lesions. MSI lesion is a kind of tumor in which the failure of mismatch repair genes (MMR), causes errors in short tandem repetitive DNA sequences known as microsatellites [260–262]. About the 5% of metastatic colorectal patients had MSI or deficient MMR [262].

Assessment of MSI status in colorectal cancer patients has prognostic and therapeutic effects. It has been shown that patients with MSI have longer OS compared to patients without MSI [263]. The reason is due to the fact that lesions with MSI have greater numbers of tumor-infiltrating lymphocytes that are activated and cytotoxic [263], so that the detection of MSI in a patient with colorectal cancer is a positive prognostic feature, particularly among young patients [263].

Today, it is clear the role of the host immune system in controlling tumor progression and new biomarkers have been included as a tool for the prediction of prognosis and response to therapy. MSI colorectal tumors display highly up-regulated expression of multiple immune checkpoints, including Programmed Death 1 (PD-1), Programmed Death-ligand 1 (PD-L1) and CTLA-4. It has been theorized that strategies involving the blockade of these immunoregulatory mechanisms might be selectively effective in this critical subset of patients [264–271].

Knowing the patients MSI status is critical since it should be correlated to immunotherapy response or resistance to fluorouracil-based therapies [272]. However, the diagnosis of MSI status is obtained by polymerase chain reaction (PCR) and immunohistochemistry achieved on pathological tissues from biopsies or surgical procedures. Therefore, it is mandatory to introduce a non-invasive and cost-effective procedure to assess MSI status. Golia Pernicka et al [273], by evaluating 254 radiomics features obtained by CT studies of 198 patients (134 patients without MSI and 64 with MSI tumors) developed 3 prognostication models based on clinical data alone, radiomics data alone, and combined radiomics and clinical data. The combined model outperformed the other two, with an AUC of 0.80 and 0.79 for the training and validation set, respectively [273]. Similar results were obtained by Fan et al. in the assessment of 119 stage II tumor patients [274]. Six radiomics features, obtained from pre-treatment CT studies, and 11 clinical data were utilized for predicting MSI status model. The combined model achieved the overall best performance obtaining an AUC, sensitivity, and specificity of 0.752, 0.663, and 0.841, respectively [274].

Wu et al. [275] developed a model by using several features to evaluate the diagnostic accuracy of dual-energy CT for discriminating MSI from MSS colorectal cancer. The AUC of the model provided relatively high diagnostic accuracy with an AUC value of 0.886, sensitivity 81.6%, and specificity of 81.6%.

At the best of our knowledge no studies assessed liver colorectal metastases MSI status and radiomics and the studies evaluating primary lesions should be interpreted with caution since the results are based on a limited number of patients. Further multicentric studies are required using a larger sample size in order to introduce this approach into everyday clinical practice [276–279].

## Mucinous histological subtypes and radiomics

With regard to histological subtypes, there are inadequate data on the role of histological subtypes in colorectal cancer patient outcomes [128]. The most common histological type is adenocarcinoma not otherwise specified (NOS), followed by mucinous adenocarcinoma, which represents 5–15% of all lesions. A greater number of BRAF and KRAS mutations and higher rate of MSI characterize mucinous subtype, so that, compared to the NOS type, the mucinous lesion is correlated to a higher risk of metastases, worse OS and a weakness response to standard chemotherapy [280–282].

In this contest, it is evident that a proper detection and characterization of liver mucinous metastases allows a better patient selection to avoid unnecessary therapies.

At the best of our knowledge, few studies have assessed the ability of Radiomics features, obtained by CT or MRI, in mucinous liver metastases characterization [105, 108, 283–285]. Granata et al evaluated radiomics data obtained from CT studies of untreated patients and from MRI studies of pre-surgical patients compared to mucinous subtype [105, 108, 283–285].

With regard to data obtained from MRI studies with hepatospecific contrast agent (EOB) [105], the univariate analysis showed a variable number of metrics, which allow to discriminate mucinous subtype: 15 significant features extracted from T2W SPACE; 13 extracted from the arterial phase; 12 extracted from the portal phase; 12 extracted from the EOB-phase. The best results at univariate analysis were reached by the wavelet_LLH_glcm_JointEntropy extracted by T2W SPACE sequence with accuracy of 92%, a sensitivity of 83%, a specificity of 94%, a PPV and a NPV of 78 and 95%, respectively, with a cut-off value of 4.61. Linear regression model increased the performance obtained respect to the univariate analysis. The best results were obtained by a linear regression model of 15 significant features extracted by the T2W SPACE sequence with accuracy of 94%, a sensitivity of 92%, a specificity of 95%, a PPV and a NPV of 83 and 98%, respectively. This study has some limits: (1) small sample size, although the analysis was done on homogeneous subset and considering all lesions; (2) the retrospective nature, (3) a manual segmentation so as (4) the impact of chemotherapy on the results [105]. The main advantage was related to the assessment of all protocol study sequences [105].

## Colorectal liver metastases growth pattern and radiomics

The majority of liver metastases have one of three common distinct histopathological growth patterns (HGPs), known as desmoplastic HGP, pushing HGP or replacement HGP, and two rare HGPs [286]. These HGPs are distinguishable because the interface between the tumor and the surrounding normal parenchymal is distinct in each growth pattern [286]. Moreover, the distinct topography of cancer cells in each HGP predicts HGP-specific interactions with parenchymal (hepatocytes and cholangiocytes) and non-parenchymal cells (sinusoidal endothelial cells, stellate cells and immune cells). However, despite these clear differences in the biology of these metastases, the molecular drivers of the distinct HGPs remain unknown. It is also currently unclear whether these distinct HGPs require different clinical management strategies, since these different patterns have shown to have prognostic significance [286]. Both Van den Eynden et al and Nielsen et al assessed the impact of the HGPs on OS in patients with liver metastases. In both studies, the desmoplastic HGP represented superior OS [287, 288]. The replacement HGP indicates not only worse OS but also resistance to systemic therapy [289]. Moreover, the HGP sub-types have diverse immune-phenotypes that are correlated to different responses to immunotherapy. Evidence showed a lower immune cells or inflammatory cells infiltration rate in the replacement sub-type while desmoplastic type is frequently surrounded by many lymphocytes [289].

The gold standard for HGP diagnosis is the histopathological analysis of the untreated resected specimen [289]. Considering the low fraction of primarily resectable metastases and the wide use of preoperative chemotherapy, clinical relevance is limited [289–295]. Therefore, a non-invasive strategy is needed to improve the prognosis and facilitate the treatment strategy [289]. In addition, a non-invasive approach would also allow for longitudinal response evaluation during treatment [289, 296–306].

Several researches have assessed the role of radiomics in the HGP pattern [109, 113, 141, 143, 145, 307], showing that HGPs of liver metastases can be effectively characterized using CT or/and MRI- radiomics data. Cheg et al [143] evaluated 126 metastatic patients who had undergone CT studies and surgical resection with histopathologically confirmed HGPs (desmoplastic HGP in 68 patients and replacement HGP in 58). The authors showed that combined, clinical-radiomics signature had the best performance in differentiating replacement from desmoplastic type [143]. Han et al. [141] assessed MRI radiomics features of 182 resected untreated metastases (desmoplastic HGPs in 59 patients and replacement HGPs in 123), using a decision tree algorithm and a combined clinical and radiomics model. They found that the combined model had good discriminating capability, with the AUC of 0.971, 0.909, and 0.905, respectively, in the training, internal validating, and external validating set [141]. Granata et al [307], analyzing a training set of 51 patients (121 metastases) and an external validation set of 30 patients (30 lesions), obtained 851 radiomics features from MRI studies in pre-surgical phase. They showed that, at univariate analysis, the best results to discriminate expansive versus infiltrative HGPs were obtained by the wavelet_LHH_glrlm_ShortRunLowGray Level Emphasis from portal phase. In addition, by using a linear regression model, the performance was higher for all sequences except that for hepatobiliary phase. Furthermore, using pattern recognition approaches, the diagnostic performance increased again and the best classifier was a weighted KNN trained [307].

The main limits of assessed studies are related to the retrospective nature, the type of segmentation and the sample size.

## Prospects and limits

**Fig. 1:**
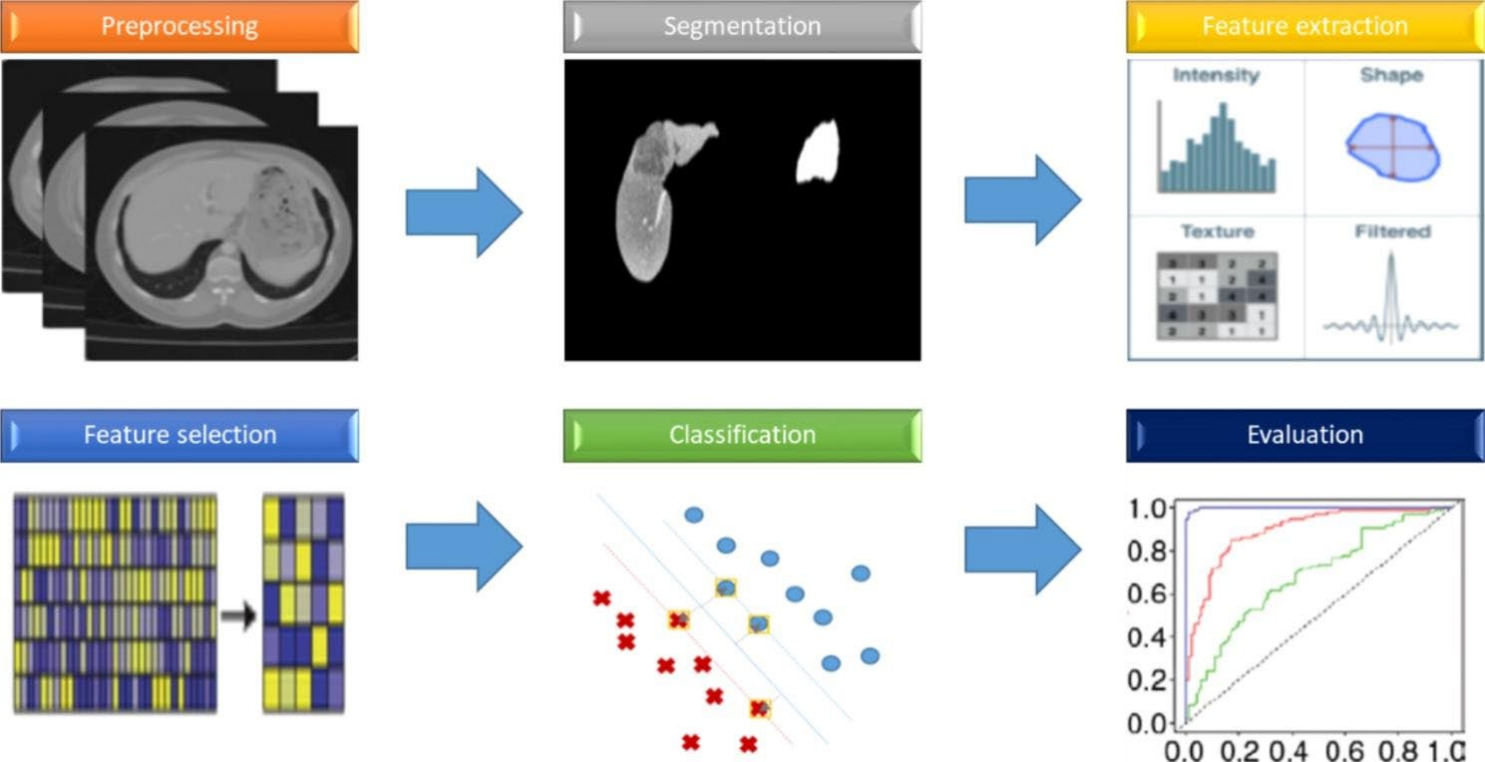
Graphic representation of a radiomics workflow

Recently, the radiomics field has developed rapidly, and the pattern recognition approaches introduction has supported faster quantitative data extraction processes [308–310]. By extracting a great deal of quantitative features from conventional medical imaging, radiomics analysis (Fig. 1) allows to obtain biological information without invasive approach. Compared with conventional and qualitative analysis, this approach should, hypothetically, improve cancer diagnosis, grading and staging, treatment response and prognosis prediction, providing professional guidance for treatment planning [311–316].

Even if there is a great interest on Radiomic as a very promising tool, however the poor standardization and generalization of studies results limit the translation of this analysis into clinical practice. Clear limits are data-quality control, reproducibility, repeatability, generalizability of results, and issues related to model overfitting [317–321].

The main critical issues are the necessity of images of high quality, so as the standardization of studies protocol and reconstruction algorithms [322, 323]. In addition, ample size and comprehensiveness of datasets, separate training and validation sets, class imbalances and overfitting are critical points. Beyond randomized trials, class inequalities are usual. So that, not only overall accuracy but also class wise accuracy, or sensitivity or specificity, should be assessed [322, 323]. When a model is not well balanced in terms of function approximation, one may encounter overfitting or, to a lesser degree, underfitting. Overfitting is due to the assessment of a large number of input parameters, which not are all relevant. To avoid overfitting, it is necessary to apply smoothing model feature, or to reduce the number of input features by reducing the number of model parameters required. Validation using a separate dataset helps detect overfitting. Underfitting arises or when a model is not able to appropriately classify data in the training and validation datasets, or if it is excessively simplistic [324–334].

Therefore, radiomics analysis should be performed considering all these aspects in order to obtain robust and reproducible data which could be generalized in other patient classes.

## Conclusion

Recently, radiomics analysis application has improved rapidly, with a consequent growing interest in the oncological field. In addition, the possibility to analyse with faster processes large amount of data with pattern recognition approaches is significantly changing the idea of ​​radiology.

In the liver metastases clinical setting, the possibility to assess mutational status (RAS or MSI), the tumour growth pattern and the histological subtype (NOS or mucinous) allows a better patient selection to avoid unnecessary treatment. Although, radiomics analysis in a non-invasive and repeatable way, also during treatment, by using imaging tools used in clinical practice, however features as the poor standardization and generalization of clinical studies results limit the translation of this analysis into clinical practice. Clear limits are data-quality control, reproducibility, repeatability, generalizability of results, and issues related to model overfitting.

## Data Availability

All data are available in the manuscript and at https://zenodo.org/record/7741988#.ZBNQm3bMK3A.
