# Correction: Colorectal liver metastases patients prognostic assessment: prospects and limits of radiomics and radiogenomics

**DOI:** 10.1186/s13027-023-00508-9

**Published:** 2023-05-05

**Authors:** Vincenza Granata, Roberta Fusco, Sergio Venanzio Setola, Roberta Galdiero, Nicola Maggialetti, Renato Patrone, Alessandro Ottaiano, Guglielmo Nasti, Lucrezia Silvestro, Antonio Cassata, Francesca Grassi, Antonio Avallone, Francesco Izzo, Antonella Petrillo

**Affiliations:** 1 Division of Radiology, “Istituto Nazionale Tumori IRCCS Fondazione Pascale – IRCCS di Napoli”, Naples, Italy; 2 Medical Oncology Division, Igea SpA, Napoli, Italy; 3 Italian Society of Medical and Interventional Radiology (SIRM), SIRM Foundation, Via della Signora 2, Milan, 20122 Italy; 4 Department of Medical Science, Neuroscience and Sensory Organs (DSMBNOS), University of Bari “Aldo Moro”, Bari, 70124 Italy; 5 Division of Epatobiliary Surgical Oncology, Istituto Nazionale Tumori IRCCS Fondazione Pascale—IRCCS di Napoli, Naples, 80131 Italy; 6 Clinical Sperimental Abdominal Oncology Unit, Istituto Nazionale Tumori, IRCCS Fondazione G. Pascale, Napoli, 80131 Italy; 7 Division of Radiology, “Università degli Studi della Campania Luigi Vanvitelli”, Naples, 80138 Italy

**Correction to**: *Infectious Agents and Cancer (2023) 18:18*

10.1186/s13027-023-00495-x

Following publication of the original article [1], the authors reported incorrect information in the ‘Data availability’ section.

The statement in the ‘Data availability’ section originally read: All data are available in the manuscript.

The statement should read: All data are available in the manuscript and at link https://zenodo.org/record/7741988#.ZBNQm3bMK3A.

The original article [1] has been updated.
